# Improved health-span and lifespan in mtDNA mutator mice treated with the mitochondrially targeted antioxidant SkQ1

**DOI:** 10.18632/aging.101174

**Published:** 2017-02-15

**Authors:** Irina G. Shabalina, Mikhail Yu. Vyssokikh, Natalia Gibanova, Robert I. Csikasz, Daniel Edgar, Anne Hallden-Waldemarson, Zinaida Rozhdestvenskaya, Lora E. Bakeeva, Valeria B. Vays, Antonina V. Pustovidko, Maxim V. Skulachev, Barbara Cannon, Vladimir P. Skulachev, Jan Nedergaard

**Affiliations:** ^1^ The Department of Molecular Biosciences, The Wenner-Gren Institute, Stockholm University, SE-106 91 Stockholm, Sweden; ^2^ The Belozersky Institute of Physico-Chemical Biology, Lomonosov Moscow State University, 119992, Moscow, Russian Federation; ^3^ Institute of Mitoengineering, Moscow State University, 119992, Moscow, Russian Federation; ^4^ Present address: Buck Institute for research on aging, Novato, CA 94945, USA

**Keywords:** reactive oxygen species, oxidative stress, mitochondria, cardiolipin, thermogenesis, brown adipose tissue, longevity

## Abstract

MtDNA mutator mice exhibit marked features of premature aging. We find that these mice treated from age of ≈100 days with the mitochondria-targeted antioxidant SkQ1 showed a delayed appearance of traits of aging such as kyphosis, alopecia, lowering of body temperature, body weight loss, as well as ameliorated heart, kidney and liver pathologies. These effects of SkQ1 are suggested to be related to an alleviation of the effects of an enhanced reactive oxygen species (ROS) level in mtDNA mutator mice: the increased mitochondrial ROS released due to mitochondrial mutations probably interact with polyunsaturated fatty acids in cardiolipin, releasing malondialdehyde and 4-hydroxynonenal that form protein adducts and thus diminishes mitochondrial functions. SkQ1 counteracts this as it scavenges mitochondrial ROS. As the results, the normal mitochondrial ultrastructure is preserved in liver and heart; the phosphorylation capacity of skeletal muscle mitochondria as well as the thermogenic capacity of brown adipose tissue is also improved. The SkQ1-treated mice live significantly longer (335 versus 290 days). These data may be relevant in relation to treatment of mitochondrial diseases particularly and the process of aging in general.

## INTRODUCTION

As a cause for the decreasing health status that accompanies aging, mitochondrial deterioration has been repeatedly suggested [1-4]. Particularly, it has been discussed that an accumulation of errors in mitochondrial DNA (mtDNA) replication would lead to mitochondrial dysfunction, including increased production of reactive oxygen species (ROS) that may both further deteriorate the mitochondria and affect the function of the rest of the cell [5-9].

However, the significance of ROS for the aging process has been doubted [10, 11], particularly based on observations in the mtDNA mutator mice [12-14]. These mice accumulate errors in their mtDNA and demonstrate subsequent alterations in their respiratory chain composition [15]. They also demonstrate an early occurrence of characteristics normally associated with aging, and they die at a young age. However, there has been no convincing evidence that oxidative damage causes these problems; rather an absence of oxidative damage has been reported [13, 16-19], but see [20].

Experimentally, an alternative avenue to examine the possible involvement of ROS in the development of aging characteristics would be to examine the ability of mitochondrially targeted antioxidants to ameliorate the health problems associated with experimentally induced aging. In this paper, we find that the mitochondrially targeted antioxidant 10-(6′-plastoquinonyl)decyltri-phenylphosphonium cation (SkQ1) [21] substantially counteracts the acquisition of aging characteristics in the mtDNA mutator mice. We also find that parameters for oxidative damage not earlier examined (cardiolipin depletion and accumulation of hydroxynonenal protein adducts) are diminished by SkQ1 treatment. These data and other antioxidant data (mitochondrially targeted catalase [22] and N-acetyl-L-cysteine treatment [23]) clearly indicate that ROS production and oxidative damage are substantial factors in the development of aging characteristics in the mtDNA mutator mice.

As the presently reluctance to associate mitochondrial dysfunction with aging through ROS and oxidative damage are largely based on the notion that these phenomena were apparently not involved in aging in mtDNA mutator mice [19, 24, 25], and as our present data indicate the opposite to be the case, our observations may also be of significance for discussions of the nature of aging and the possibility to ameliorate the aging process therapeutically.

## RESULTS

In order to examine the significance of possible enhanced ROS production for the development of early aging characteristics in the mtDNA mutator mice, we have here followed such mice that were treated or not with SkQ1. In the following, we first describe phenotypic manifestations of the SkQ1 treatment and we then examine the effect of such a treatment on markers of oxidative damage. We also examine the effect of SkQ1 treatment on total lifespan in mtDNA mutator mice.

### SkQ1 treatment attenuated the manifestation of kyphosis and alopecia in mtDNA mutator mice

**Figure 1:**
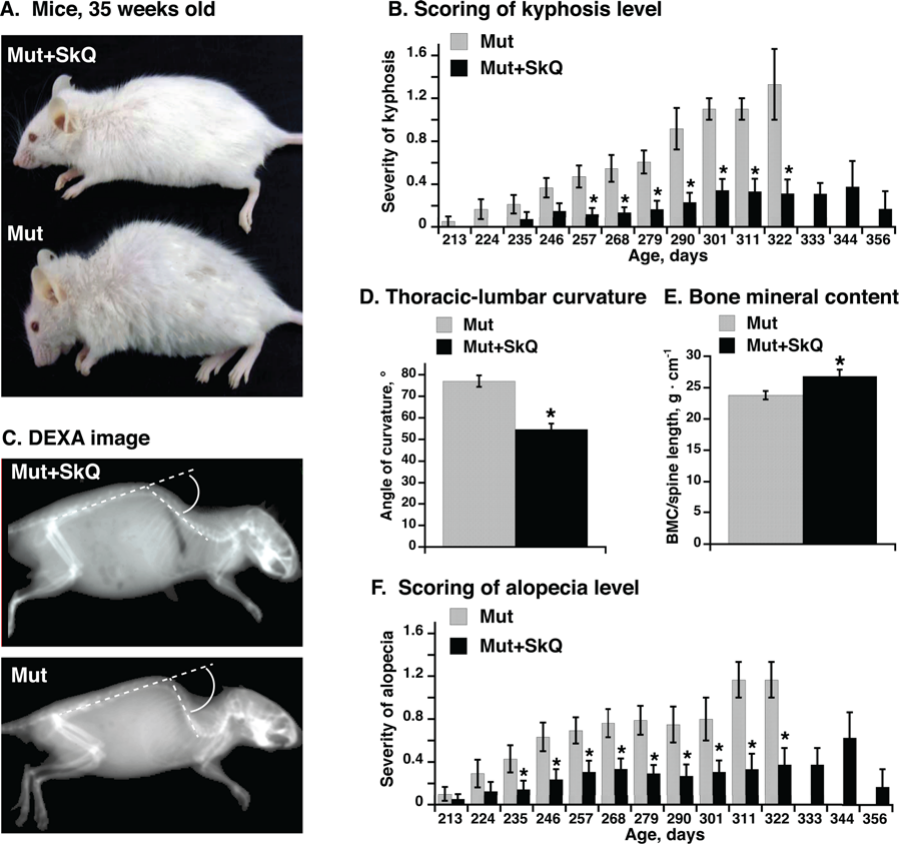
Effect of SkQ1 treatment on appearance, kyphosis and alopecia in mtDNA mutator mice (**A**) Pictures of mtDNA mutator mice: a non-treated (Mut) mouse and an SkQ1-treated (Mut+SkQ1) littermate female mouse, 248 and 256 days of age respectively. (**B**) Scores of kyphosis manifestation (arbitrary units) in mtDNA mutator mice. Each bar represents the mean ± S.E. score from mice of both genders: 21 Mut (10 females + 11 males) and 22 Mut+SkQ1 (13 f + 11 m) in ages 213-268 days; 11 Mut (5 f + 6 m) and 13 Mut+SkQ1 (6 f + 7 m) in ages 268–290 days; in ages 290–356 days, the number of mice decreased with age according to survival; the lowest number of mice in a group was 4 (2 f + 2 m). Day number under each bar indicates the center day of 11 days of mouse age. (**C**) X-ray picture of a non-treated mouse and an SkQ1-treated littermate female mtDNA mutator mouse (290 ± 4 days old). White circle arc indicates curvature angle measurements. Note that the angle is calculated from the extended line of the lower spine. (**D**) Angle of thoracic-lumbar curvature determined as in C. Each bar represents the mean ± S.E. from 10–11 mice of both genders in each group; Mut, 287 ± 7 days old, 5 f + 6 m; Mut + SkQ1, 284 ± 6 days old, 6 f + 5 m. (**E**) Bone minerality of spine cord area from scapula to lumbar. Each bar represents the mean ± S.E. from 6–8 female mice in each group. Mut, 290 ± 11 days; Mut + SkQ1, 287 ± 9 days. (**F**) Scores of alopecia manifestation in mtDNA mutator mice. The mice are the same as those in (B). * in B-F indicates a statistically significant difference between non-treated and SkQ1-treated mtDNA mutator mice (p < 0.05).

Already at an age of less than 230-250 days, mtDNA mutator mice show features normally associated with aging, as exemplified with the ≈250 days old mtDNA mutator mouse in Fig. 1A which confirms observations originally published by [12, 13]. Strikingly, treatment of these mice with the SkQ1, not from birth but only from an age of ≈100 days, markedly diminished this phenotype, resulting in mice presenting a mouse much more similar to wild-type mice of this age (Fig. 1A). These dramatic improvements were quantitatively analyzed in the ensuing graphs.

One feature of the aging process is the occurrence of (hyper)kyphosis, i.e. (over)curvature of the spine [12, 13]. The severity of this malfunction has been manually scored in Fig. 1B every 11^th^ day (as exemplified in Supp. Table S1). Whereas hyperkyphosis developed gradually but consistently in the non-treated mtDNA mutator mice, the occurrence was low and fairly stable in the SkQ1-treated age-matched mice, even though the SkQ1-treated mice lived longer (see below) and thus could be followed to a higher age. Hyperkyphosis can be quantitated through X-ray analysis. Fig. 1C illustrates such a measurement, performed on mice of equal age. The mean values of these measurements are compiled in Fig. 1D. As seen, there was a significant effect of SkQ1 in diminishing the degree of kyphosis.

**Table 1:** Phospholipids and fatty acyl composition in mitochondria from wild-type mice [SkQ1-treated (WT+SkQ1) or non-treated (WT)] and mtDNA mutator mice [SkQ1-treated (Mut+SkQ1) or non-treated (Mut)]

Mitochondria from tissue	Phospholipid and their fatty acyls (FA)	WT	WT+SkQ1	Mut	Mut+SkQ1
Skeletal muscle	Phosphatidylcholine	51.0±3.6	50.0±2.8	52.0±4.2	52.0±3.1
	Phosphatidylethanolamine	19±2	18±1	22±2	19±2
	*Cardiolipin*	**17±1**	**20±2**	**13±1#**	18±1*
	Phosphatidylinositol	5.0±0.7	4.0±0.6	5.0±0.6	4.0±0.4
	Phosphatidylserine	5.0±1.1	5.0±0.9	4.0±0.8	4.0±0.7
	Phosphatidic acid	3.0±0.6	3.0±0.2	4.0±0.4	3.0±0.3
	*Saturated FA*	**20±1**	**20±2**	**31±2#**	23±1*
	Monounsaturated FA	22±2	20±1	21±1	22±0
	Polyunsaturated FA n-3	22±1	24±1	24±1	22±1
	*Polyunsaturated FA n-6*	**37±3**	**37±1**	**24±1#**	33±1*
Liver	Phosphatidylcholine	45.5±4.2	44.2±2.6	47±3	44.5±3.8
	Phosphatidylethanolamine	34±2	32±2	35±2	33±3
	*Cardiolipin*	**15±2**	20±2*	**12±1**	18±1*
	Phosphatidylinositol	5.0±1.1	3.0±1.0	5.0±0.7	4.0±0.9
	Phosphatidylserine	0.5±0.2	0.8±0.2	1.0±0.5	0.5±0.1
	*Saturated FA*	**19±1**	**21±1**	**29±2#**	22±2*
	Monounsaturated FA	24±1	22±1	22±1	23±1
	Polyunsaturated FA n-3	21±1	23±2	26±2	24±1
	*Polyunsaturated FA n-6*	**36±2**	**35±2**	**23±2#**	32±1*

The values are expressed in mol % and represent the means ± S.E. of 6 independent mitochondrial preparations isolated in parallel from 4 groups of mice, 252–259 days old.

* indicates statistical difference between SkQ1-treated and non-treated mice (p < 0.05).

# indicates statistical difference between wild-type and mtDNA mutator mice (p < 0.05).

Measurement of bone minerality of the spinal cord from scapula to lumbar revealed a higher bone mineral content of this area in the SkQ1-treated mtDNA mutator mice (Fig. 1E).

Non-treated mtDNA mutator mice showed severe alopecia (hair loss) [12, 13]. This pathology was much less evident in mice treated with SkQ1 (Fig. 1F). It may especially be noted that these cohorts of mice were single-caged. Thus, the alopecia and the loss of whiskers were mouse-autonomous effects. Earlier studies have kept mice in larger groups where mouse-mouse interaction (particularly between males) may substantially affect the outcome, leading to exaggerated fur loss (barbering). – As fur protects against heat loss [26] and as the mice were living at 22°C, i.e. below their thermoneutral zone, the implication is that the SkQ1-induced amelioration of the fur status is not only a general indication of less rapid advancement of aging features but also that it can diminish the heat loss and thus the risk for hypothermia [27].

### SkQ1 treatment prevented loss of fat and improved the estrus state in mtDNA mutator mice

**Figure 2:**
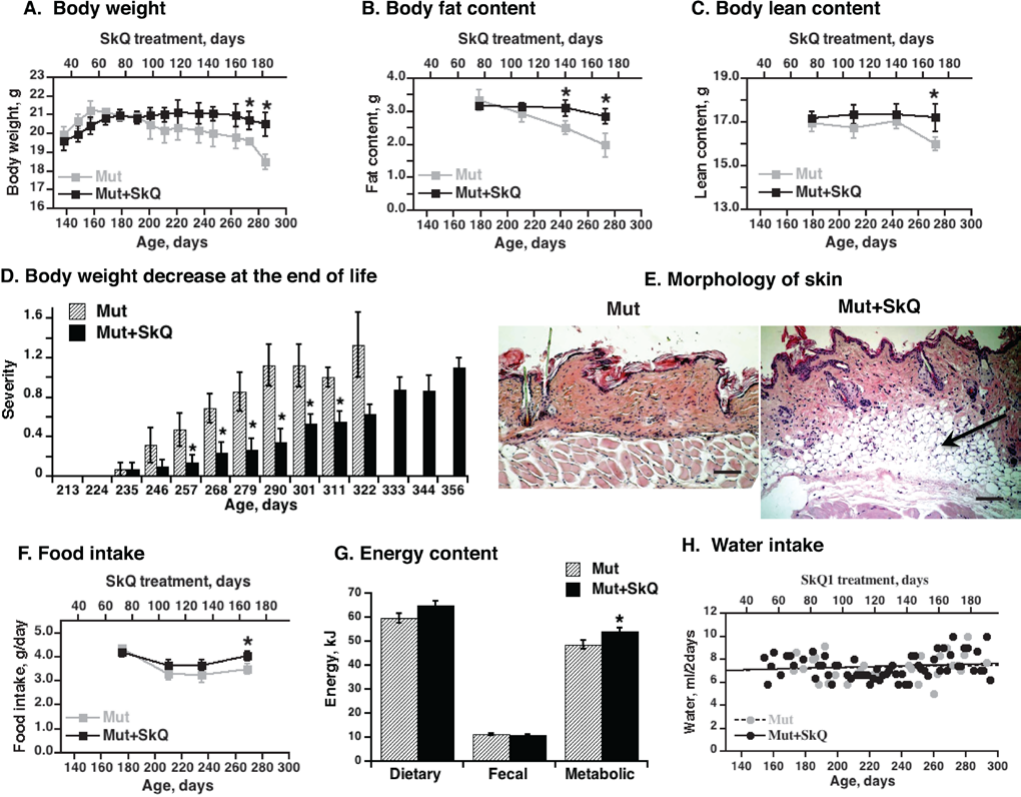
Effects of SkQ1 treatment on body energy stores and intake in mtDNA mutator mice (**A**) Body weight, (**B**) body fat content and (**C**) lean body mass as a function of age (± 7 days). The points are means ± S.E. In these “paired death” experiments, 8 female mice were in each group until age 238 days; after this, the number of mice decreased with time, depending on survival, with 4 mice in each group at the final point. (**D**) Scoring of acute body weight decrease in mtDNA mutator mice. The mice are the same as in Fig. 1B. (**E**) Skin morphology. The skin samples were from the back region of non-treated and SkQ1-treated littermate female mice of the same age (290 ± 4 days). Bar is 100 μm. The subdermal fat region is indicated by arrow. (**F**) Food intake. The mice are the same as in Fig. 2A. (**G**) Dietary, fecal and metabolic energy in mtDNA mutator at age 250 ± 7 days. Metabolic energy values were obtained by subtraction of fecal energy from dietary energy consumed. * in A-D and F indicates a statistically significant difference between non-treated and SkQ-treated mtDNA mutator mice (p < 0.05, n = 8 mice in each group). (**H**) Water intake in mtDNA mutator mice treated or not with SkQ1. The points were obtained by combining remaining water from all mice in each group (8 female mtDNA mutator mice) and subtracting this from the total amount of water supplied (values per mouse).

The SkQ1-treated mtDNA mutator mice showed no decrease with age in body weight and body fat content. Both these parameters significantly decreased in the non-treated mice (Fig. 2A, B). Lean body mass was kept at stable levels longer than fat mass, but from an age of ≈270 days, it decreased in the non-treated mtDNA mutator mice (Fig. 2C). SkQ1 treatment prevented this loss of lean body mass (Fig. 2C). Acute body weight loss is an important criterion for the running assessment of mouse health (Supp. Table S1). As seen on Fig. 2D, this parameter was much improved in the SkQ1-treated mice.

In mtDNA mutator mice of 290 days, there were nearly no subdermal lipid stores left whereas the stores were well maintained in the SkQ1-treated mice (Fig. 2E). During the time when the weight curves for the non-treated and SkQ1-treated mice diverged, the food intake and metabolic energy consumption in the SkQ1-treated animals was slightly larger than that of the non-treated mtDNA mutator mice (Fig. 2F and G). This was probably secondary to the generally better status of the SkQ1-treated mice. Addition of SkQ1 to the drinking water did not change the water intake (Fig. 2H).

**Figure 3:**
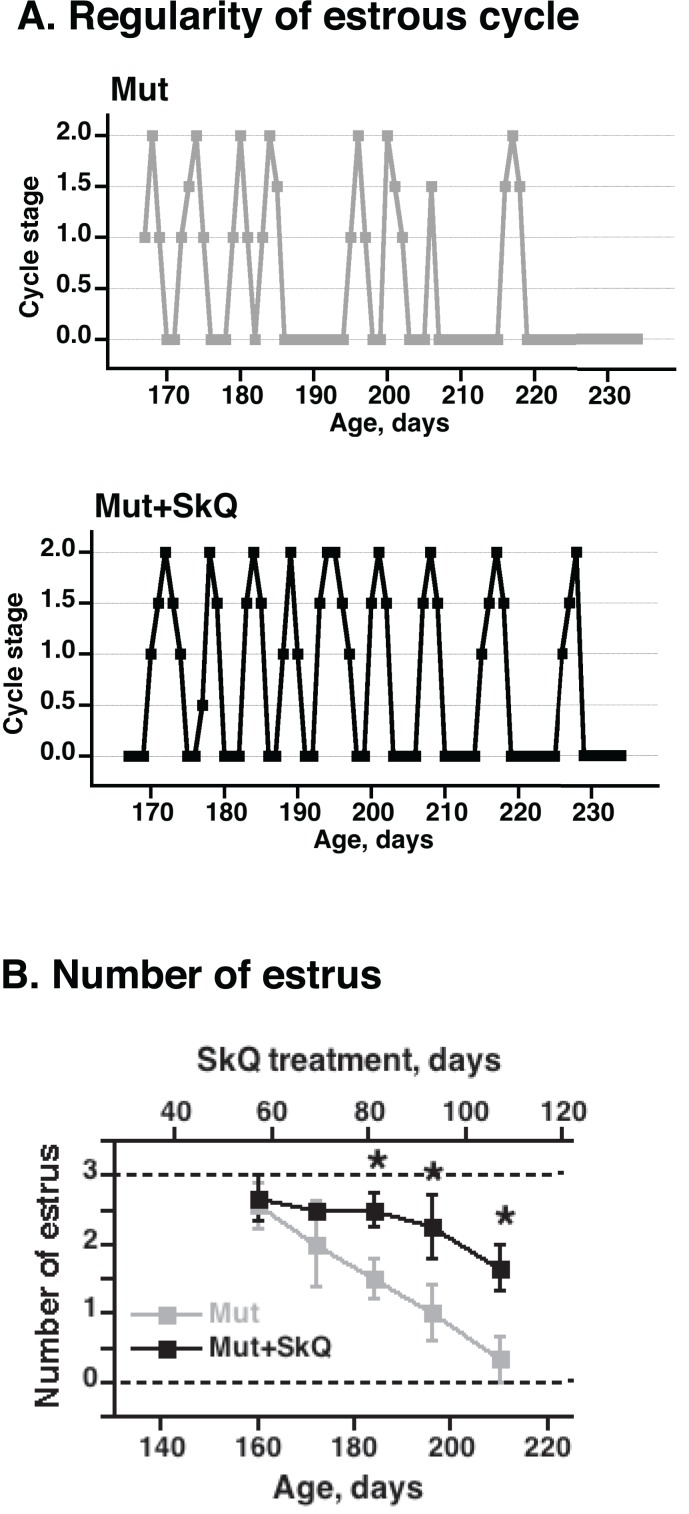
Effects of SkQ1 treatment on estrus cycle in mtDNA mutator mice (**A**) Examples of graphs of estrus cycles in non-treated and SkQ-treated mtDNA mutator female mice. Examination of estrus cycle was done by light microscopy of vaginal smears. Estrus was characterized by the presence of large cornified cells with degenerated nuclei and was indicated as estrus stage 2 on the graphic presentation. Diestrus was identified by presence of leucocytes and mucous and was indicated as 0 on graphs. Proestrus and metestrus were characterized by the presence of nucleated epithelial cells or leucocytes together with cornified cells and were indicated on the graphs as intermediate stages 0.5 – 1.5. (**B**) Number of estruses during 12 days measured as a function of age (± 7 days). The points are means ± S.E. of 7-8 female mice for each group. * indicates a statistically significant difference between non-treated and SkQ1-treated mice (p < 0.05).

Through leptin signaling [28], body lipid stores may affect ovulation in the mice. Accordingly, non-treated mtDNA mutator mice exhibited a successively impaired estrus cycle with irregularity and loss of estrus (Fig. 3). The SkQ1 treatment increased the number of estruses and ameliorated the irregularity of the estrus cycle (Fig. 3).

### Positive effects of SkQ1 treatment could be attributed to its antioxidant properties

The data collected above demonstrate that treatment of mtDNA mutator mice with SkQ1 significantly delays the aging characteristics observed in the mtDNA mutator mice. As SkQ1 was originally developed as a mitochondrially targeted antioxidant [21], we examined whether the treatment of mtDNA mutator mice with SkQ1 would affect ROS-related parameters that may be linked to SkQ1's beneficial effects.

In the typical lipid peroxidation process, ROS will interact with omega-6 and omega-3 fatty acids (mainly linoleic acid and arachidonic acid) in the membrane phospholipids, leading to the release of malondialdehyde (MDA) and 4-hydroxynonenal (4-HNE) that may form adducts with proteins. We examined whether these parameters were affected by the SkQ1 treatment.

**Figure 4:**
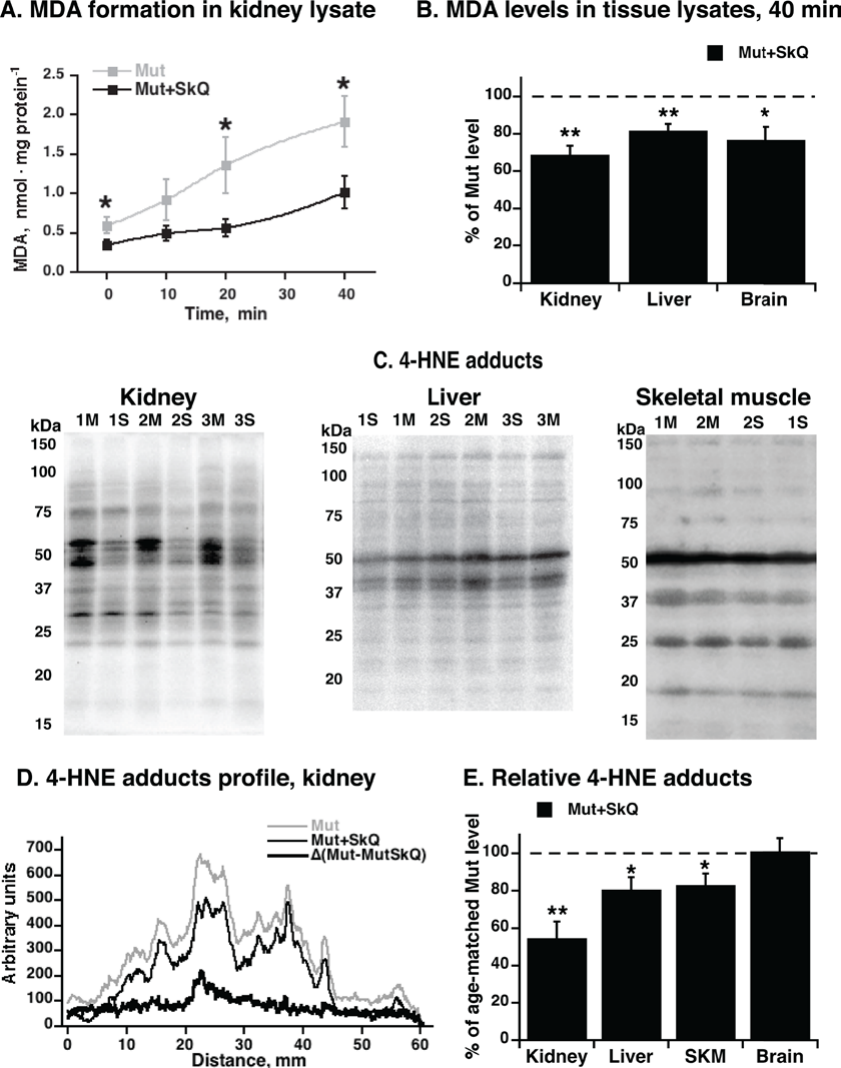
MDA formation and content of 4-HNE adducts in tissues from mtDNA mutator mice non-treated and treated with SkQ1 (**A**) MDA formation in kidney lysate exposed to normal atmospheric oxygen concentration and pressure at 37°C. (**B**) Levels of MDA formed in liver, brain and kidney lysates after 40 min of exposure of tissue to normal atmospheric oxygen concentration and pressure. (**C**) Representative immunoblot analysis of 4-hydroxynonenal (4-HNE) adducts in kidney (*left panel*), liver (*middle panel*) and gastrocnemius skeletal muscle (*right panel*) tissue lysate from non-treated (M) and SkQ1-treated (S) mtDNA mutator mice (15 μg protein/lane). Numbers 1, 2, 3 indicate age-and gender-matched samples. Validation of the assay is presented in Fig. S1A. Loading control was performed by Ponceau Red staining (shown for kidney in Fig. S1C). (**D**) Profile of 4-HNE-adducts in kidney lysate. (**E**) Relative 4-HNE-adducts in kidney, liver, skeletal muscle (SKM) and brain tissue lysates. Tissue samples were collected in parallel from non-treated and SkQ1-treated mice (268–300 days old, both genders) in the paired death experimental setup. In B and E, for each pair, the mean level of 4-HNE-adducts in the non-treated mouse was set to 100 % (indicated as dashed line) and the amount in the paired (age- and gender-matched) SkQ1-treated mouse was expressed relative to this. The means ± S.E. for 4-7 mice are shown. * and ** in A, B and E indicate statistical significance between SkQ1-treated and non-treated mice (p < 0.05 and p < 0.01, respectively).

We followed the formation of MDA in kidney lysates (Fig. 4A). The rate of formation was markedly lower in the lysates from the SkQ1-treated mtDNA mutator mice. We made similar observations in lysates from liver and brain (Fig. 4B). These experiments thus indicated that at least *in vitro*, tissues obtained from SkQ1-treated mice were less prone to lipid peroxidation than tissues from non-treated mice.

To examine whether similar differences occurred *in vivo*, we estimated the endogenous level of lipid peroxidation by analysis of the *in vivo* formed 4-HNE adducts to cellular proteins. A lower content of 4-HNE-adducts was observed in kidney samples from SkQ1-treated mice as compared with the level in kidneys of non-treated mice (Figs. 4C - E). Also in liver and skeletal muscle, 4-HNE adducts were significantly (although less markedly) lower in SkQ1-treated mice (Fig. 4C - E); in brain, no difference was observed (Fig. 4E). These findings are in line with our data that the *in vivo* administrated SkQ1 accumulates in brain in much smaller amount than in kidney, liver and skeletal muscle (A. Andreev-Andreevsky et al., in preparation).

As one of the main sources of the released MDA and 4-HNE are the polyunsaturated fatty acids of the mitochondrial membrane phospholipids [29], we examined the effect of SkQ1 on the phospholipid composition of mitochondria from different tissues. This experiment is exemplified for skeletal muscle and liver in Table 1. As seen in Table 1, neither the mutation nor the treatment with SkQ1 had any marked effect on the content of most phospholipid classes. However, one phospholipid class, cardiolipin, was relatively decreased in the mtDNA mutator mice as compared to wild-type mice. This effect was revealed in both skeletal muscle and liver (Table 1). Further, the SkQ1-treatment restored the cardiolipin amount in the mtDNA mutator mice to wild-type levels in the mtDNA mutator mice (Table 1).

We further analyzed the fatty acyl composition of the total phospholipids of the mitochondrial membranes. The content of polyunsaturated n-6 fatty acids (PUFA n-6) in mtDNA mutator mice was markedly decreased to only 2/3rd of wild-type levels both in skeletal muscle and liver mitochondria of mtDNA mutator mice (Table 1). The lowering of polyunsaturated fatty acids level was compensated for by saturated fatty acids. The SkQ1 treatment of the mtDNA mutator mice fully prevented this remodeling, such that wild-type levels were retained.

As cardiolipin generally contains 4 linoleyl (18:2, n-6) moieties per molecule, it is likely that the decrease in cardiolipin amount (by ≈5 mol %) is largely responsible for the loss of n-6 PUFAs (by ≈10 mol %), and that the retention of the n-6 PUFAs by SkQ1 treatment is due to the preservation of cardiolipin in the SkQ1-treated mtDNA mutator mice.

### SkQ1 normalized mitochondrial ultrastructure in liver and heart

Cardiolipin is believed to be important for mitochondrial structure and function [32]. We therefore examined whether the large changes in cardiolipin amount were associated with alterations in these parameters.

**Figure 5:**
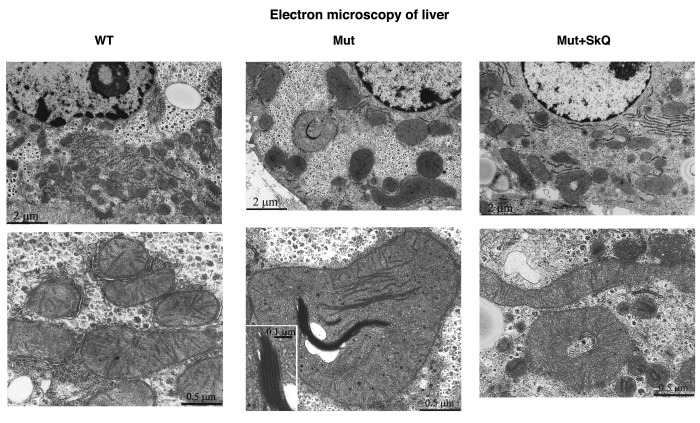
Liver mitochondrial structure Electron micrographs of liver from wild-type mice (left panel), mtDNA mutator mice (middle panel) and mtDNA mutator mice treated with SkQ1 (right panel). Animals were 245 – 252 days old. Insertion in the middle panel, lower micrograph: intramitochondrial myelin-like structure at higher magnification. Similar findings were observed in 4-5 other mice of each group.

Fig. 5 shows electron microscopic pictures of liver cells from wild-type mice, mtDNA mutator mice and SkQ1-treated mtDNA mutator mice. The mitochondria in mtDNA mutator mice seemed larger than those in wild-type mice. Moreover, some of the mitochondria in the mtDNA mutator mice contained elongated electron-dense inclusions composed of series of membranes packed in a myelin-like manner. The SkQ1 treatment prevented both these changes.

**Figure 6:**
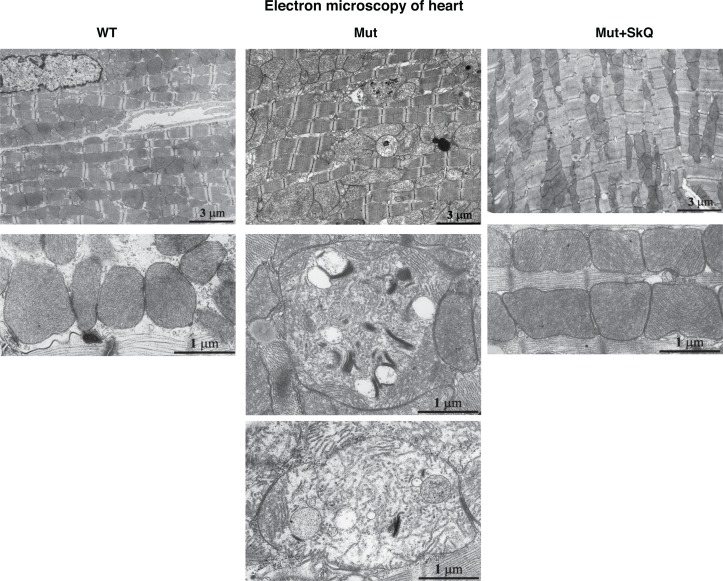
Heart mitochondrial structure Electron micrographs of heart from wild-type mice (left panel), mtDNA mutator mice (middle panel) and mtDNA mutator mice treated with SkQ1 (right panel). Animals were 245 – 252 days old. Similar findings were observed in 4-5 other mice of each group.

Safdar *et al*. [33] observed similar mitochondrial alterations in skeletal and heart muscles of mtDNA mutator mice. We confirmed this observation in heart muscle. Also in this tissue, SkQ1 prevented disorganization of the mitochondrial ultrastructure (Fig. 6). Moreover, the number of mitochondria per μm^2^ of heart muscle was increased by SkQ1 from 100 ± 5 % to 128 ± 6 % (P < 0.05) in the wild-type mice and from 69 ± 3 % to 125 ± 6 % (P < 0.001) in the mtDNA mutator mice. SkQ1 did not influence the area occupied by the inner mitochondrial membrane in the wild-type mice but prevented 42 % (P < 0.01) decrease in this area observed in the mtDNA mutator mice.

### SkQ1 improved the function of isolated mitochondria

**Figure 7:**
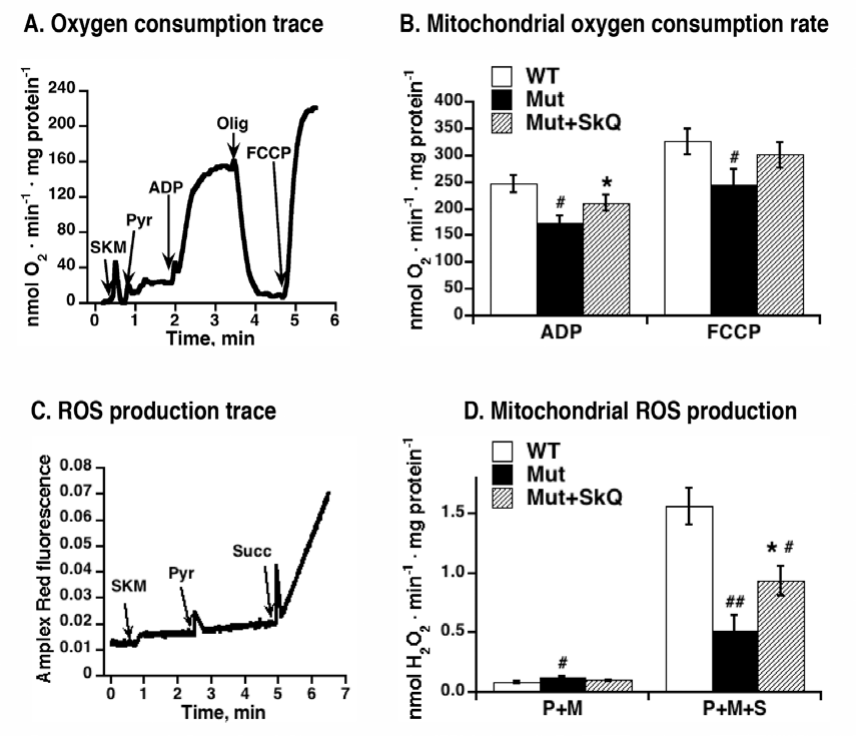
Effect of SkQ1 treatment on function of isolated mitochondria (**A**) Example of an oxygen consumption trace in mitochondria isolated from skeletal muscle of mtDNA mutator mouse. Additions were 0.25 mg skeletal muscle mitochondria (*SKM*), 5 mM pyruvate (*Pyr*), 250 μM ADP, 2 μg /ml oligomycin (*Olig*) and 1.4 μM FCCP. (**B**) Rates of oxygen consumption in mitochondria isolated from skeletal muscle of SkQ1-treated and non-treated mtDNA mutator mice. Analysis was performed as shown in 7A. (**C**) Amplex Red fluorescence in intact mitochondria isolated from skeletal muscle of mtDNA mutator mouse. Additions were 0.25 mg skeletal muscle mitochondria (*SKM*), 5 mM pyruvate (*Pyr*), 5mM succinate (*Succ*). Malate (3 mM) was present in the medium. The analyses were performed in the same mitochondrial preparations as in (A) in parallel with the oxygen consumption measurements. (**D**) Rates of hydrogen peroxide production in mitochondria isolated from skeletal muscle of wild type mice (WT), SkQ1 non-treated (Mut) and treated (Mut + SkQ1) mtDNA mutator mice. Analysis was performed as shown in C. *P+M* indicates the presence of complex I substrates (pyruvate + malate) and *P+M+S* indicates the presence of three substrates (pyruvate + malate + succinate). In A and D, the values represent the means ± S.E. of 6 independent mitochondrial preparations isolated in parallel from treated and non-treated groups of mice of an age of 252 – 259 days. * in A-D indicates statistical difference between SkQ1-treated and non-treated mice (p < 0.05).

In the mtDNA mutator mice, there is a significant decline of ADP- and FCCP-inducible respiration of liver and heart mitochondria, as compared to wild-type mitochondria [15]. To examine whether SkQ1 treatment would affect the development of this mitochondrial deterioration, we analyzed the oxygen consumption of skeletal muscle mitochondria isolated from SkQ1-treated and non-treated mtDNA mutator mice. Fig. 7A exemplifies the bioenergetic kinetics observed in such experiments. As summarized in Fig. 7B, ADP-stimulated respiration was higher in mitochondria from SkQ1-treated mtDNA mutator mice than in mitochondria from mtDNA mutator mice. There was no observable effect on state 4 respiration (i.e. after oligomycin) but there was a tendency to a higher maximal respiratory capacity (i.e. after addition of the artificial uncoupler FCCP). Thus, both ultrastructural and bioenergetic features of mitochondria from mtDNA mutator mice were ameliorated by SkQ1 treatment.

### Higher mitochondrial ROS production in SkQ1-treated mice reflects an improved respiratory chain capacity

Hydrogen peroxide production resulting from oxidation of a complex I substrates (pyruvate+malate) or of mixed complex-I and complex-II substrates (pyruvate +malate+succinate) was measured in skeletal muscle mitochondria from non-treated and SkQ1-treated mtDNA mutator mice, as exemplified in Figs. 7C and D. Hydrogen peroxide production supported by complex I substrates alone was not affected by SkO1-treatment (Fig. 7D). However, on mixed substrates hydrogen peroxide production rate in isolated mitochondria from SkQ1-treated mice was higher than in mitochondria from non-treated mice (Fig. 7D). This effect of SkQ1 may be the result of an improved respiratory electron transfer activity and/or more directly from a higher membrane potential in the mitochondria of SkQ1-treated mice. It is shown that the ROS production that results from reverse electron flow from exogenously provided succinate is augmented by a higher membrane potential [34, 35, 36, 37]. As to the *in vivo* SkQ1 treatment, it does not lead to increased general oxidative damage (4-HNE adduct formation was actually decreased following SkQ1 treatment, see Fig. 4E).

### SkQ1 treatment improves thermogenic capacity of brown adipose tissue

**Figure 8:**
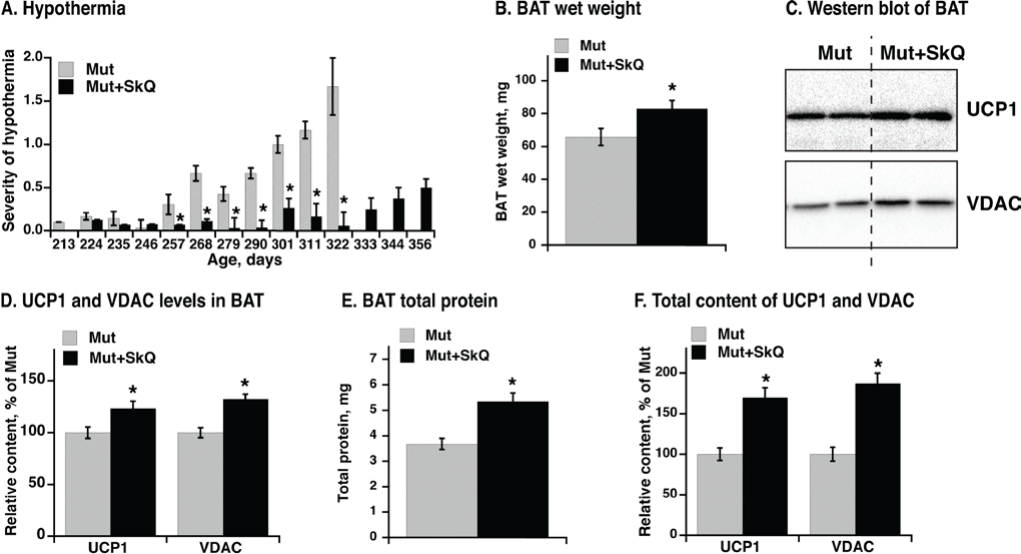
Effect of SkQ1 treatment on thermogenesis and brown adipose tissue of mtDNA mutator mice (**A**) Scoring of manifestations of hypothermia (based on body temperature, shivering and posture position) in SkQ1-treated and non-treated mtDNA mutator mice. Gender and number of mice in each group are as in 1B. (**B**) Wet weight of interscapular brown adipose tissue (BAT). (**C**) Western blots analyses of VDAC and UCP1 in BAT (tissue homogenate protein, 10 μg per lane). (**D**) Relative concentration of mitochondrial proteins. Western blots as in C were quantified. The mean level of UCP1 and VDAC in control mice was set to 100 % and the levels of protein in BAT from SkQ1-treated mice expressed relative to this. (**E**) Total protein content per BAT depot. (**F**) Total content of UCP1 and VDAC per mouse. Content of each protein per μg homogenate protein estimated from Western blot analysis (in E) was multiplied with the total protein content of BAT (in C) from the same mouse. The values in B and D - F represent the means ± SE of 4-5 independent tissue preparations in each group, analyzed singly or in duplicate. * indicates statistical significance between SkQ1-treated and non-treated mice (p < 0.05).

Hypothermia is a characteristic feature of mtDNA mutator mice and develops markedly after about 220 days, as evaluated qualitatively in Fig. 8A. SkQ1-treated mtDNA mutator mice show almost no indication of hypothermia during this period. Additionally, whereas mtDNA mutator mice to a very high degree developed hypothermia towards the end of their life, the SkQ1-treated mtDNA mutator mice did not present with this problem even as they became moribund (Fig. 8A).

A main source for sustained regulatory heat production in mammals is brown adipose tissue. There was a significant positive effect of SkQ1 treatment on interscapular brown adipose tissue mass (Fig. 8B). Analysis of mitochondrial proteins in brown adipose tissue homogenate revealed a higher level of mitochondrial voltage-dependent anion channel (VDAC) per mg brown adipose tissue total protein (Figs. 8C, D) in SkQ1-treated mtDNA mutator mice, indicating an increase in the number of mitochondria. More functionally, there was also an increase in the amount of the rate-limiting protein for thermogenesis, i.e. uncoupling protein (UCP1) (Fig. 8C, D). The total brown adipose tissue protein was also higher in SkQ1-treated mtDNA mutator mice (Fig. 8E). For thermogenesis on whole organism level, the total thermogenic capacity is more important than concentration of proteins. Therefore we estimated the total content of VDAC (≈ amount of mitochondria) and UCP1(≈ total thermogenic capacity) in the interscapular brown adipose tissue (Fig. 8F) by multiplying the specific content of each of these proteins (Fig. 8D) with the total amount of protein (Fig. 8E). The outcome was that the total mitochondrial content and particularly the total content of UCP1 was more than 50% higher in the SkQ1-treated mtDNA mutator mice than in the non-treated mice (Fig. 8F). These observations indicate that the lack of hypothermia in the SkQ1-treated mtDNA mutator mice can largely be ascribed to an enhanced (or preserved) capacity for heat production in their brown adipose tissue.

### SkQ1 treatment delays development of mtDNA mutator mice immobility

**Figure 9:**
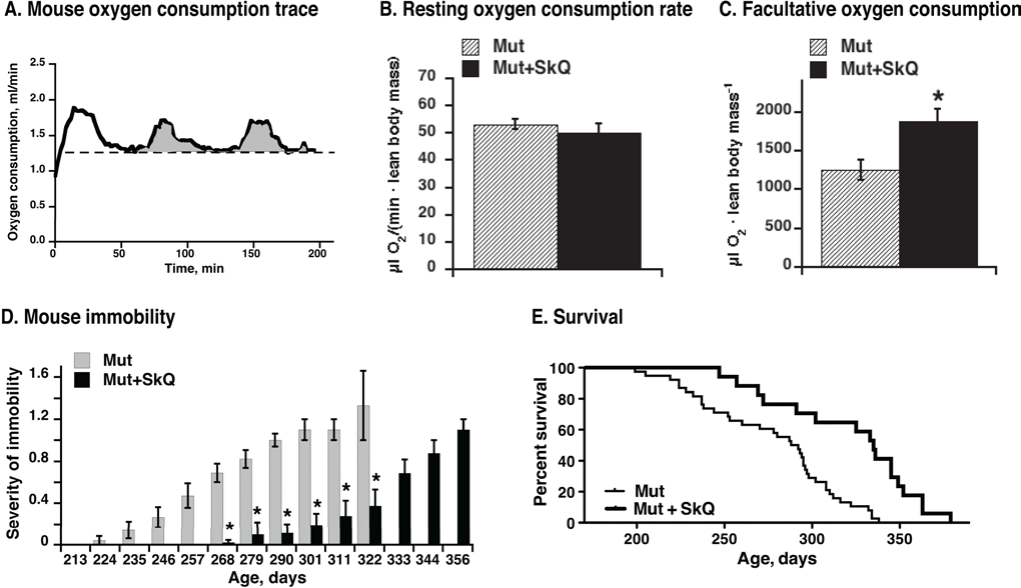
Effect of SkQ1 treatment on mouse metabolism, motility and survival (**A**) Representative trace of oxygen consumption (metabolism) of a mouse. Running oxygen consumption rate is indicated as black bold curve, the lowest (resting at 22°C environmental temperature) oxygen consumption rate indicated as a dashed straight line and the oxygen consumed above the resting rate is indicated as grey area. Note that the oxygen consumption rate at 22°C does not represent the basal metabolic rate, as the mice are examined below their thermoneutral temperature zone.(**B**) Metabolic rates in non-treated and SkQ-treated mice (≈ 200 days of age) at 22°C ambient temperature measured as shown in A. (**C**) Metabolic activity of mtDNA mutator mice. Activity was determined as amount of oxygen consumed above resting metabolic rate during two hours in the metabolic chamber (as shown in 9A). In B and C, the values represent the means ± S.E. of 6 mice in each group. (**D**) Scoring of manifestations of immobility in SkQ1-treated and non-treated mtDNA mutator mice. (**E**) Survival curves of mtDNA mutant mice non-treated (thin line) or treated with SkQ1 (thick line), n= 38 (15 males and 23 females) in the non-treated group and n= 17 (6 males and 10 females) in SkQ1-treated group. No gender dependence on life-span was observed (not shown). Mice were single caged. Mean survival time for the non-treated group was 277 ± 6 days, for the SkQ1-treated 321 ± 10 days (+ 16%); median lifespan was 290 days for non-treated mice and 335 days (+ 16%) for SkQ1-treated mice. Comparison of survival curves with log-rank (Mantel-Cox) test yields p < 0.0001 and with Gehan-Breslow-Wilcoxon test yields p = 0.0012.

Oxygen consumption of mice at their living temperature 22°C (Fig. 9A) reflects mouse general activity inclu-ding adaptive thermogenesis. The SkQ1-treated mtDNA mutator mice exhibited higher metabolism (oxygen consumption rates) above the resting rates (Fig. 9B and C), probably related to visibly higher mobility of animals (Fig. 9D), better retention of mitochondrial oxidative phosphorylation (Fig. 7B) and improved brown adipose tissue thermogenic capacity (Fig. 8F).

### Significance of improved thermogenic capacity and physical activity on cause of death in SkQ1-treated mtDNA mutator mice

In Table 2, we have collected descriptions of phenotypic features of mtDNA mutator mice treated and non-treated with SkQ1 at the point of euthanasia. It may be noted that in the non-treated mtDNA mutator mice, more than 50 % of the mice displayed phenotypes related to body temperature regulation problems: low body temperatures, piloerection, shivering. In the SkQ1-treated mice, these features were not at all prominent. Thus, the SkQ1 treatment retained mitochondrial bioenergetic capacity, both in muscle and in brown adipose tissue, thereby eliminating a major cause of death. In the SkQ1-treated group, anemia and body weight decrease became the prominent features in the moribund mice.

**Table 2:** Incidence of symptoms in mtDNA mutator mice at the point of euthanasia during the survival experiment

mtDNA mutator mice		mtDNA mutator mice + SkQ1	
*Symptoms*	*Number (and %) of mice in the group from total 28*	*Symptoms*	*Number (and %) of mice in the group from total 13*
Keeping posture position; shivering	25 (89%)	Severe paleness	9 (69%)
Body temperature below 34°C	23 (82%)	Mouse hides, lies still but is startled when touched	8 (62%)
Chronic body weight loss by 15% during several weeks	21 (75%)	Chronic body weight loss by 15% during several weeks	8 (62%)
Severe piloerection	20 (71%)	Mouse is unable to stand up on back legs; dis-coordination, asymmetry of movement	5 (38%)
Severe anus prolapse and bleeding	6 (21%)	Keeping posture position; shivering	5 (38%)
Soft feces, diarrhea	5 (18%)	Severe dehydration	5 (38%)
Acute body weight loss (2-3 g during 1 week)	5 (18%)	Severe anus prolapse and bleeding	3 (23%)
Severe paleness	4 (14%)	Soft feces, diarrhea	3 (23%)
Mouse is unable to stand up on back legs; dis-coordination, asymmetry of movement	3 (11%)	Acute body weight loss (2-3 g during 1 week)	3 (23%)
Severe dehydration	2 (7%)	Severe piloerection	1 (8%)
Mouse hides, lies still but is startled when touched	1 (4%)	Body temperature below 34°C	1 (8%)

The heavy line separates the symptoms seen in >50 % of all mice from those seen in <50 %.

Postmortem examination also revealed positive effects of SkQ1 treatment (Table 3). Particularly, the heart pathology (Fig. S2A) often observed in the mtDNA mutator mice [12, 22] was much rarer (Table 3). Liver (Fig. S2B), as well as kidney (Fig. S2C), pathology was milder, and the hydrocephalus observed occasionally in the mtDNA mutator mice was not found in the SkQ1-treated mice (Table 3). In contrast, certain other pathological findings occurred irrespectively of SkQ1 treatment: cecum and colon displayed pathological changes in all animals in both groups, and particularly the spleen was also pathological in nearly all animals, irrespective of SkQ1 treatment or not (Table 3). The spleen pathology is probably related to the paleness observed to a high extent in the SkQ1-treated mice during their last days (Fig. S2D). It would simply seem that the SkQ1-treated mtDNA mutator mice lived so much longer than the non-treated mice that anemia had time to develop sufficiently to become life-threatening.

**Table 3:** Histopathological findings at necropsy of mtDNA mutator mice untreated or treated with SkQ1

Organ	Histopathology	Mut n = 11	Mut+SkQ1 n = 11
Heart	1) The myocardium exhibited moderate myocardial degeneration, characterized by irregularly shaped nuclei and rich cytoplasmic vacuolation in myocytes	5	2
	2) Perivascular or interstitial fibrosis and Anitschkow cells	2	0
Brain	Mild internal hydrocephalus	3	0
Kidney	The renal cortex displayed occasional dilated tubules and increased cytoplasmic basophillia in the epithelial lining an occasional proximal tubule - characteristics of nephropathy: mild moderate	4 5	6 0
Liver	1) Chronic degenerative changes characterized by enlarged periportal hepatocytes having granular cytoplasm or large and small fatty vacuoles. Portal areas displayed moderate fibrosis and hyperplastic oval cells at sites: 1a) mild 1b) moderate	1 5	2 0
	2) Degenerative changes with abnormal nuclear features (pseudo-inclusions)	3	0
	3) The sinusoids exhibited scattered, small aggregates of hematopoietic cells or/and myelopoetic cells	5	5
Spleen	1) The red pulp displayed extensive hematopoetic activity	10	11
	2) The white pulp displayed small lymphatic follicles	9	9
Cecum and the proximal colon	Severe mucosal hyperplasia with chronic catarrhal inflammation. Epithelium degeneration, necrosis, apoptosis. Infiltration leukocytes predominately macrophages. Mucosal atrophy coexisted with sites of the mucosa thickened by hyperplasia, forming plague-like or irregular papillary projections lined by crowed, tall columnar, basophilic cells with numerous mitotic figures.	11	11

11 mice in each group were analyzed in the survival experimental setup.

### SkQ1 treatment increased the lifespan of mtDNA mutator mice

It is implicitly evident from several of the above data series that in addition to the general positive effect that SkQ1 had on the general health of the mtDNA mutator mice, SkQ1 also prolonged the life of these mice. This is directly shown in Fig. 9F. The first mouse from the non-treated group died at an age of 199 days whereas the first SkQ1-treated mouse lived for 70 days longer. The median survival time for the non-treated group was 290 days; for the SkQ1-treated group it was 335 days, an increase in life expectancy of 45 days or 16 %.

Thus, the SkQ1-treated mtDNA mutator mice not only increased their life-span; they also experienced a healthier life, with prolonged estrus cycles, adequate weight maintenance and less pathology.

## DISCUSSION

In the present study, we have demonstrated that treatment of mtDNA mutator mice with the mitochondrial targeted antioxidant SkQ1 ameliorates most of the syndromes associated with the mitochondrial polymerase γDNA proof-reading malfunction in these mice. Thus, the occurrence of hair loss, kyphosis, loss of estrus cycle, body weight loss, reduced lipid stores, hypothermia, immobility and torpor-like states were delayed and did not become so marked. Pathological signs of heart, liver and kidney malfunction were diminished. Functionally, our data suggest that SkQ1 counteracts free radical oxidation of polyunsaturated fatty acids, primarily in the cardiolipin class of phospholipids. This results in a slight but statistically significant difference in formation of MDA and 4-HNE between the control group and the animals treated with SkQ1. This in turn diminishes the levels of lipid peroxidation products and protein modifications. Preserving mitochondrial cardiolipin is likely the reason for protection of intact mitochondrial ultrastructure, required for normal bioenergetic function of mitochondria. Functional mitochondria in brown adipose tissue and skeletal muscle are required for the production of sufficient heat to protect the mice from hypothermia. Probably mainly due to this heat production, SkQ1-treated mtDNA mutator mice survive substantially longer than non-treated mtDNA mutator mice. Taken together, our observations indicate that mitochondrial oxidative damage is a substantial cause in the acquisition of aging characteristics in the mtDNA mutator mice. Since present reluctance to accept ROS as a causative agent of aging characteristics is largely based on the concept that increased oxidative damage had not been observed in the mtDNA mutator mice, and as our present results clearly demonstrate that counteraction of oxidative damage ameliorates aging in the mtDNA mutator mice, the above-described data are also important for discussions concerning the molecular background of the aging process. A further issue is whether SkQ1 or SkQ1-like substances are of therapeutic interest in relation to mitochondrial diseases in general and aging in particular.

### A mechanism of action for SkQ1

The experiments presented here point to a series of events that could largely explain the ameliorating effect of SkQ1 on the health of mtDNA mutator mice. There are several issues to be discussed concerning the suggested mode of action.

SkQ1 is an antioxidant and was developed as such to counteract oxidative damage in mitochondria [21]. However, it is a controversial issue as to whether there is increased oxidative stress (and damage) in mtDNA mutator mice, and an antioxidant should only work if there is increased oxidative stress. Initial studies of the mtDNA mutator mice indicated that there was no evidence (e.g. in the form of carbonylation) that the mitochondria of these mice displayed significantly increased oxidative damage [13, 16, 17]. However, this point has weakened over the years [20]. Particularly, direct measurement of hydrogen peroxide in the mitochondria indicated that in the mtDNA mutator mice an increased hydrogen peroxide level could be detected with age [38], and there are several indirect observations that are most easily explained by in increased ROS exposure in these mitochondria [22,23]. To this should evidently be added the observations that we report here, of decreased cardiolipin and polyun-saturated fatty acid levels in the mtDNA mutator mice.

We observed changes in cardiolipin levels in mtDNA mutator mice and reversal of these changes by SkQ1. Under oxidative stress, cardiolipin is peroxidized earlier than any other phospholipids [39] and its decomposition is prevented by SkQ1 *in vitro* [40]. Cardiolipin protection is probably the primary target for SkQ1 operating as an antioxidant [41, 42]. In the mitochondria, the SkQ1 cation may combine directly with the cardiolipin anion [41].

It may be suggested that the mitochondria in the mtDNA mutator mice truly produce slightly more ROS than mitochondria in wild-type mice [38]). However, the increase is rather small so that it to a large extent is scavenged by the easily oxidized polyunsaturated fatty acids mainly found in the mitochondrial cardiolipin, leading to the formation of 4-HNE that then forms adducts with (mitochondrial) proteins. Thus, the ROS levels may never rise to such levels that other signs of oxidative stress become measurable. The problems caused by cardiolipin diminishment and formation of 4-HNE protein adducts may be sufficient to explain most of the negative effects observed; at least, as seen here, when cardiolipin levels *in vivo* are protected, mitochondrial competence is markedly improved.

### A therapeutic role for SkQ1 and SkQ1-like compounds in mitochondrial diseases and aging

The mtDNA mutator mice do show mitochondrial damage akin to that occurring in some mitochondrial diseases, and the ability of SkQ1 to ameliorate the effect points to a possibility to ameliorate some mitochondrial diseases by treatment with antioxidants. Indeed, antioxidant treatments such as mitochondrially overexpressed catalase [22] and supplying large amounts of N-acetyl cysteine [23] have been shown to be beneficial against mitochondrial problems. The advantage of SkQ1, as well as of MitoQ [43], are that they are mitochondrially targeted antioxidants that can be directly supplied in relatively small amounts. The present studies could therefore promote further attempts to use such compounds therapeutically.

A classical hypothesis for aging was that mitochondrial damage would result in increased ROS production that in its turn would increase mitochondrial oxidative damage that then in a deleterious cycle, would augment itself [8]. However, this idea has lost general acceptance in recent years. A not insignificant reason for this has been reports from studies of the mtDNA mutator mice that the mitochondria seemingly do not display enhanced ROS production or enhanced oxidative damage. However, studies such as the present one do imply that oxidative damage may be important for the aging characteristics observed, at least in the mtDNA mutator mice, and these studies may thus resurrect the ROS hypothesis for aging. We found increased longevity in the SkQ1-treated mtDNA mutator mice. Although these mice may correctly be considered an artificial model, the marked effects of SkQ1 treatment would imply that counteracting ROS damage could be one factor leading to prolonged life. There are indeed indications that treatment with targeted antioxidants such as SkQ1 not only are effective against specified diseases or in artificial models, but may also prolong lifespan in general, in wild-type animals [21, 44, 45].

## MATERIALS AND METHODS

### Animal breeding, maintaining and treatment

All animal experiments were performed at the Wenner-Gren Institute, Stockholm University. MtDNA mutator animals were obtained by intercrossing of mice heterozygous for the *PolgA^mut^*allele, developed as described [12]. The mice were genotyped as previously described [12]. For the present studies, homozygous mutant (PolgAmut/PolgAmut) mice were used. When wild-type mice were studied, they were homozygotes for the wild-type; heterozygotes were not used in the present experiments. Mice were successively recruited to the study, as they became available. The mice had been backcrossed to C57Bl/6 for at least 7 generations, but as the PolgA gene is located on chromosome 7 at the 45.04 cM location close to the tyrosinase gene determining black/white color [46], the mice were either black or white. The mice were fed ad libitum (R70 Standard Diet, Lactamin AB, Vadstena, Sweden), had free access to water, and were kept on a 12:12 h light:dark cycle at 22°C.

For treatment with SkQ1, the mice were singly housed from an age of ≈100 days when also the treatment started. The experimental mice were treated with SkQ1 dissolved in ethanol and added to the drinking water provided in 30 ml volume in a small bottle; the water was changed every second day. The final concentration of ethanol was 0.1 %; this ethanol concentration was also present in the water of the control (non-treated) mice. SkQ1 doses (taking into account absorbance of SkQ1 on the inner surface of the bottle) were 0.7-0.8 μmol/(day · kg body weight) for males and 0.9-1.0 μmol/(day · kg) for females.

Several experimental designs were used, as specified in the figure legends. In the “survival” experiments, the mice were followed until they were moribund (defined as the day when the General Condition score (see below) exceeded 2.5), at which time the mice were euthanized, according to the requirements of the ethical committee. Some of these mice underwent postmortem analysis performed at the Swedish State Veterinary Institute to establish the cause of death and general health. In the ”simultaneous” set-up, mice (6 from each group) were killed at a time where a clear distinction in health had developed between the SkQ1-treated and non-treated mtDNA mutator mice (i.e. at an age of ≈250 days, after 150 days of SkQ1 treatment). In the “paired death” experiments, one age- and sex-matched mouse from the SkQ1-treated group was killed when one mouse in the non-treated group became moribund (8 females and 5 males from each group).

Animal protocols were in accordance with the guidelines for the humane treatment of animals and were reviewed and approved by the Animal Ethics Committee of the North Stockholm Region.

### Monitoring of physiological parameters, metabolic rate and DEXA

From an age of about 140 days, body temperature, body weight, body fat content, lean body mass, food and water intake and estrus cycle were monitored. The body temperature was measured with a rectal probe for mice (RET-3 plugged to amplifier BAT-12, Physiterm Instruments Inc., NJ, USA). Measurements of body weight and body temperature were made every 10-14 days, always at the same time (14.00-14.30). Magnetic resonance relaxometry (EchoMRI^TM^ Whole Body Composition Analyzer, Echo Medical Systems, LLC, Texas, USA) was performed every month to measure live body composition in terms of fat and lean tissue content. For food intake studies, *ad libitum* food intake of individually housed mice was measured over a period of 5 consecutive days every month. Known excess amounts of food were added on the first day, and leftover food was measured after 5 days for each mouse individually. To determine net energy intake, diet and fecal samples were analyzed for energy content, using adiabatic bomb calorimetry (Oxygen Bomb Calorimeter 6300, Parr Instrument, USA). Water intake was measured over periods of 2 days.

For monitoring metabolic rates, oxygen consumption was measured by indirect calorimetry (INCA system, Somedic, Hörby, Sweden). The mouse remained in its own cage with food and water, and the cage was placed in a constant airflow in a temperature-controlled chamber. Oxygen consumption measurements were performed for 3 hours (light period, 22°C). Metabolic rate at 22°C (“resting metabolic rate”) was defined as the average of the stable lowest level of oxygen consumption for at least 10 minutes. Facultative (additional) metabolism (mainly related to a physical activity) was estimated as the total area above the lowest metabolic rate during the last 2 hours (the mice were agitated during the first hour in the metabolic chamber and this period was not used for estimation of this parameter).

Determination of the reproductive status of the female mice was made by microscopic examination of unstained vaginal secretion smears. The proportion between cell types was used for the determination of estrus cycle phases [47].

The carcasses of some killed mice were placed at −20°C and used for densitometer dual energy X-ray absorptiometry (DEXA) assay to measure bone minerality (assessment of osteoporosis) and to make X-ray pictures for quantitative determination of kyphosis. DEXA was performed using PIXImus small animal DEXA system (Lunar, Madison, WI, USA).

### Survival curve and scoring of phenotype features and symptoms of poor welfare

Every week during the entire experiment, the mice were assessed and scored for features of aging phenotype (signs of alopecia, kyphosis) and for clinical signs of poor welfare [paleness, immobility, discoordination with asymmetry of movement, piloerection, withdrawnness, dehydration (squared tail and lowered skin turgor), shivering, anal prolapse with bleeding, soft feces, and skin damage]. The scoring was made by a veterinarian [to whom the code of treatment was not known (blinded experiment)] together with a researcher to state the general appearance for each individual animal. Individual records of body weight, fat content and body temperature were taken into consideration for the welfare evaluation. Mild symptoms scored between 0 and 1, moderate symptoms between 1 and 2 and severe above 2. An example of a scoring table is shown in Supp. Table S1.

### Histopathological analysis

In the survival experiment, the following tissues were collected for histopathological analysis: liver, spleen, kidney, pancreas, lung, heart, brain, stomach and different parts of the digestive tract, and skin. Tissues were fixed in 10 % formalin, processed using routine procedures, and embedded in paraffin. A 5 μm section was cut from each block and stained with haematoxylin and eosin.

### Protein oxidative modification biomarker

Oxidative stress biomarker [4-hydroxynonenal (4-HNE) adducts] were analyzed in total tissue homogenates of gastrocnemius skeletal muscle, brain, liver and kidney. For collection of tissues, mice from the paired-death experimental setup [8 age- and gender-matched (268-300 days) mice (5 females and 3 males) in each group] were anaesthetized for 1-2 min by a mixture of 79 % CO_2_ and 21 % O_2_ and decapitated. Tissues were dissected out and rapidly placed in liquid nitrogen; the tissues were powdered under liquid nitrogen, weighed, divided into small amounts and stored under nitrogen gas at −80°C. A small amount was homogenized in RIPA buffer with proteinase inhibitor (Complete mini, Roche), and protein concentration was quantified using the Lowry method. 4-HNE-adducts were detected by immunoblot analysis with polyclonal antibodies from Alpha Diagnostics (HNE12-S, dilution 1:1000) as described [48]. 4-HNE-BSA protein conjugate (HNE12-C, Alpha Diagnostics) was used as an internal control. For *in vitro* 4-HNE adduct formation, wild-type skeletal muscle tissue homogenate was incubated with 64 or 640 μM 4-HNE for 20 min at 37°C in the medium used for the mitochondrial oxygen consumption measurements.

### Susceptibility to lipid peroxidation

Susceptibility to lipid peroxidation was estimated based on the kinetics of malondialdehyde (MDA) formation, principally as described [48]. MDA was assumed to be the main released substance that reacts with thiobarbituric acid [49]. In brief, tissue powdered as above was homogenized with 50 mM Tris-HCl buffer (pH 7.5). The MDA level was measured during incubation at 37°C under vortexing every 10 - 20 min. After precipitation with trichloroacetic acid, an equal volume of 0.38% (w/w) thiobarbituric acid solution in 0.25 M HCI was added to the samples. The mixture was heated at 90°C for 10 min, and then cooled on ice. Light absorbance was read at 532 nm. 1,1,3,3-tetraethoxypropane was used for calibration. Malondialdehyde was expressed as nmol MDA/mg protein.

### Immunoblotting of mitochondrial proteins

Interscapular depots of brown adipose tissue were collected from mice from the paired-death experimental setup [5 age-matched (268-300 days) females in each group]. Aliquots of homogenates were stored at −80°C after supplementation with protease inhibitor cocktail (Complete Mini, Roche Diagnostics GmbH, Mannheim, Germany). The protein concentration of the samples was quantified using the Lowry method. Western blot analysis was performed on protein extracts as described [50]. Samples were loaded on 15 % SDS-polyacrylamide gel (15 μg/lane) and run for 3 h in 1 x Tris-glycine-SDS running buffer (BioRad). Protein samples were transferred by electroblotting to a PVDF membrane (Amersham Pharmacia Biotech, Buckinghamshire, UK). UCP1 and VDAC expressions were examined using either a UCP1 antibody produced in rabbit against the C-terminal UCP1 decapeptide at a dilution of 1:3000 (in-house product) or a VDAC antibody from Cell Signaling (#4661S) diluted 1:2000.

After incubation with primary antibodies, the membranes were washed with PBST buffer and incubated with secondary anti-mouse antibodies conjugated with horseradish peroxidase (Cell Signaling Technology, Inc. Danvers, MA, USA, dilution 1:2000). The membranes were washed in PBST and incubated with detection reagents (ECL Plus Kit, Amersham Biosciences); the chemiluminescence signal was measured with a CCD (charge-coupled device) camera. The blots were quantified using the Image Gauge V3.45 program (Fuji Film, Tokyo, Japan).

### Isolation of mitochondria

Mice from the simultaneous experimental setup [6 age-matched (245 – 252 days) mice in each group] were anaesthetized for 1 min by a mixture of 79 % CO_2_ and 21 % O_2_ and decapitated. Mitochondrial preparations from SkQ1-treated and non-treated mtDNA mutator mice were directly compared on the same experimental day. Skeletal muscles from the hind limbs of one mouse were placed into ice-cold medium containing 100 mM sucrose, 50 mM KCl, 20 mM K-TES, 1 mM EDTA and 0.1 % (w/v) fatty acid-free bovine serum albumin (BSA) and were freed of white fat and connective tissue, weighed and used for mitochondrial isolation. Liver mitochondria were isolated as previously described [15]. The tissues were finely minced with scissors and homogenized in a Potter homogenizer with a Teflon pestle. During mincing and homogenizing, the skeletal muscles were treated with nagarse added to the medium at a concentration of 1 mg per g tissue. Throughout the isolation process, tissues were kept at 0-2°C.

Mitochondria were isolated by differential centrifugation. The tissue homogenates were centrifuged at 8 500 *g* for 10 min at 2°C using a Beckman J2-21M centrifuge. The resulting supernatant, containing floating fat and nagarse, was discarded. The pellet was resuspended in ice-cold medium containing 100 mM sucrose, 50 mM KCl, 20 mM K-TES, 1 mM EDTA and 0.2 % BSA. The resuspended homogenate was centrifuged at 800 *g* for 10 min, and the resulting supernatant was centrifuged at 8 500 *g* for 10 min. The resulting mitochondrial pellet was resuspended in the same buffer (but albumin-free) and centrifuged again at 8 500 *g* for 10 min. The final mitochondrial pellets were resuspended by hand homogenization in a small glass homogenizer in the buffer remaining in the pellet. The concentration of mitochondrial protein was measured using fluorescamine [51] with BSA as a standard. Mitochondrial suspensions were kept on ice for no longer than 4 h.

### Mitochondrial oxygen consumption

Skeletal muscle mitochondria (0.25 mg protein/ml) were incubated at 37°C under magnetic stirring in a buffer consisting of 100 mM sucrose, 20 mM K_+_-TES (pH 7.2), 50 mM KCl, 2 mM MgCl_2_, 1 mM EDTA, 4 mM KP_i_, 3 mM malate and 0.1 % fatty-acid-free BSA. Oxygen consumption rates were monitored with a Clark-type oxygen electrode (Yellow Springs Instruments, Yellow Springs, OH, USA) in a sealed incubation chamber at 37°C as described [52]. The output signal of the oxygen electrode amplifier was electronically time-differentiated and collected every 0.5 s by a PowerLab application program Chart v5.1.1. (AD Instruments Ltd, Oxford, UK).

### Mitochondrial ROS production

Mitochondrial H_2_O_2_ production was determined fluorometrically using Amplex Red reagent (Molecular Probes, Eugene, OR, USA). Oxidation of Amplex Red coupled with horseradish peroxidase to reduction of H_2_O_2_ results in formation of resorufin, a red fluorescent product. Mitochondria (0.16 mg mitochondrial protein ml^-1^) were incubated at 37°C under magnetic stirring in a buffer containing 100 mM sucrose, 20 mM K_+_ TES (pH 7.2), 50 mM KCl, 2 mM MgCl_2_, 1 mM EDTA, 4 mM KP_i_, 3 mM malate, 0.1 % fatty-acid-free BSA, 5 μM Amplex Red, horseradish peroxidase (12 units ml^-1^) and superoxide dismutase (45 units ml^-1^). The reaction was initiated by adding 5 mM pyruvate followed by the addition of 5 mM succinate. The increase in fluorescence emitted through a band pass filter of 600 ± 20 nm (excitation, 545 nm) was followed in a 3 ml cuvette for 10-15 min in a Sigma spectrofluorometer. The rate of H_2_O_2_ production was calculated as a change in fluorescence intensity, as described earlier [36]. Calibration curves were obtained by adding to the assay medium freshly diluted H_2_O_2_ (the concentration of stock solution was checked at 240 nm using a molar extinction coefficient of 43.6).

### Mitochondrial phospholipid analysis

Lipids were extracted from skeletal muscle or liver mitochondria (1-2 mg by protein content) isolated from mtDNA mutator mice as well as from wild-type mice (each strain treated or not with SkQ1) according to [53]. Phospholipids were separated by 2D-thin layer chromatography (TLC) on Silica gel 60 plates (Merck, Darmstadt, Germany) at 25°C in an oxygen-depleted system. All solvents and chromatography tanks were deoxygenated by nitrogen for at least 1 h. Each sample was loaded on a 12×12 cm plate in the left bottom corner. For separation in the first direction, an eluent composed of chloroform/methanol/25 % NH3 (65:35:5 per vol) was used. After front migration to the top of the plate, the plate was dried under a stream of nitrogen. As the second chromatography system, an eluent composed of chloroform/acetone/methanol/acetic acid/water (50:20:10:10:5 per vol) was used. After drying under a stream of nitrogen, phospholipids were visualized on TLC plates by staining with iodine vapour, scraped off and quantified according to [54]. Identification of single phospholipids positions was done according to migration of standards (Avanti Polar Lipids, USA), separated at the same conditions.

For analysis of the fatty acid composition, phospholipids were converted to their methyl esters through methanolysis in oxygen-free 14% methanol solution of boron trifluoride at 100°C. Fatty acid methyl esters (FAME) were separated by gas chromatography with a flame-ionization detector (GC/FID) on Hewlett-Packard 5890, using a CP Sil 88 50 capillary column with helium as carrier gas. Identification and quantification of fatty acids were made by comparison of their retention times to FAME GLC-68B standards (NuCheck Inc., USA).

### Electron microscopy

Liver and heart were collected from mice in the simultaneous experimental setup [6 age- and gender-matched (≈250 days old) mice in each group]. The pieces of tissue were fixed with a 3% glutaraldehyde solution (pH 7.4) for 2 hours at 4°C, then overfixed with 1% osmium tetraoxide solution for 1.5 hours and dehydrated in an alcohol series with increasing alcohol concentrations (70 % alcohol saturated with uranyl acetate). The tissues were embedded in an Epon-812 epoxy resin. Ultrathin sections were made with a Leica Ultracut UCT microtome (Germany) followed by lead citrate staining according to Reynolds [55]. The resulting preparations were scanned and photographed using an H-12 electron microscope (Hitachi, Japan).

### Statistics

All data are presented as means ± S.E. Differences between genotypes were examined with Student's t-test. Survival curves were analyzed using the method of Kaplan and Meier, and comparisons of survival curves were made using the log-rank (Mantel-Cox) test and the Gehan-Breslow-Wilcoxon test.

## SUPPLEMENTARY MATERIALS TABLES AND FIGURES


