# Supplementary material for: Improved health-span and lifespan in mtDNA mutator mice treated with the mitochondrially targeted antioxidant SkQ1
Source: Aging (Albany NY). 2017 Feb 15;9(2):315–36. doi: 10.18632/aging.101174 (PMC5361666; doi:10.18632/aging.101174)
Supplement: Supplementary file 1 [file aging-09-315-s001.pdf]

SUPPLEMENTARY MATERIAL

Supplementary Table S1. Scoring of mouse phenotype.

**Age, days**	**290 days**		**301 days**		**311 days**	
Symptom	value	severity	value	severity	value	severity
Porphyria		0		0		0
Weight (loss)	23.7	1.5	23.3	2.0	22.5	2.5
Diarrhea		0		0		0
Temperature (loss)	35.5	0.5	35.3	1.0	34.5	2.5
Kyphosis		1.5		1.5		1.5
Lowered activity		1.0		0.5	Disco-ordinated	2.0
Fur loss		0.5		0.5		0.5
Cleaning, grooming		0.5		0.5		0.5
Dehydration grade		1.0		1.0		1.0
Piloerection		0.5		0.5		2.5
Paleness		2.0		2.0		2.0
General condition		*1.5*		*1.5*		*2.5*

Example of part of an evaluation protocol for a single mouse. For each parameter, the scale goes to 3.0. The mice were euthanized when the General Condition value exceeded 2.5 (or any other evident reason was observed).

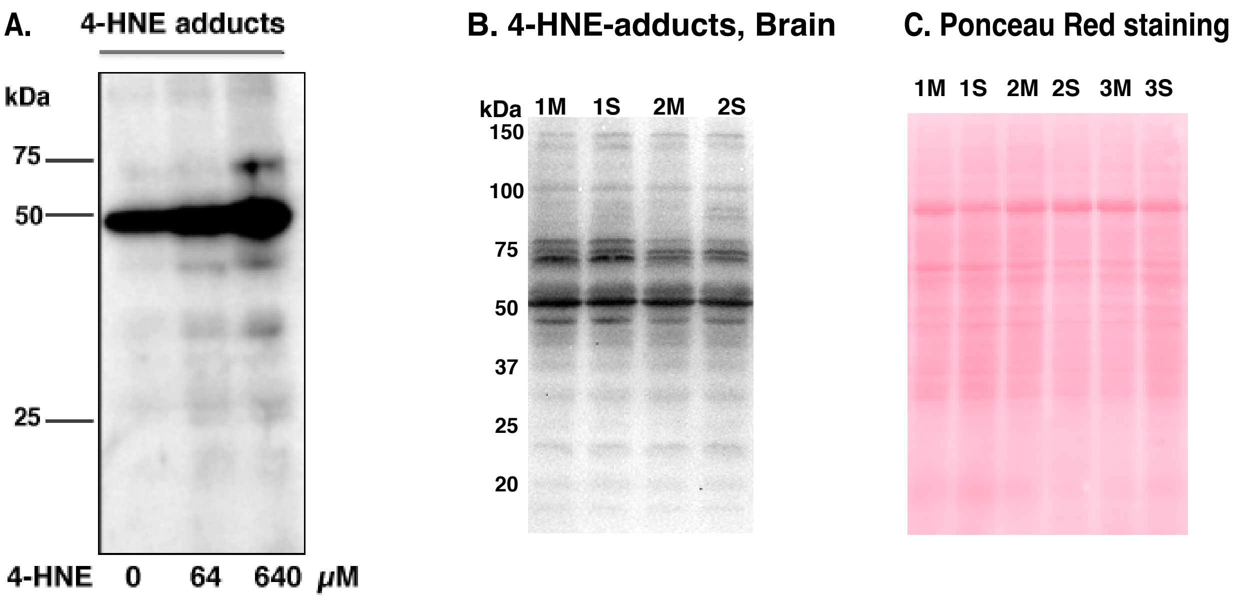

Supplementary Figure S1 (related to Fig. 4 and Table 1). 4-HNE adducts, Ponceau Red staining and TLC assay.

(A) Demonstration of in vitro 4-HNE adducts formation in wild-type skeletal muscle tissue lysate. Tissue lysates (15 µg protein/lane) were incubated with 0 or 64 or 640 µM 4-HNE for 20 min at 37 °C in the medium used for the mitochondrial oxygen consumption measurements.

(B) Representative immunoblot analysis of 4-HNE-adducts in brain lysates from non-treated (M) and SkQ1-treated (S) mtDNA mutator mice (15 µg protein/lane). Numbers 1 and 2 indicate age- and gender-matched samples.

(C) Ponceau Red staining of immunoblot membrane of kidney lysates shown in Fig. 4C. Numbers 1, 2 and 3 indicate age- and gender-matched samples.

A. Heart morphology

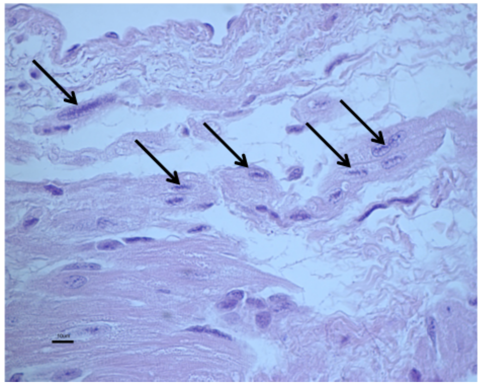

Several myocardial fibers displaying Anitchkow cell features (arrows)

B. Liver morphology

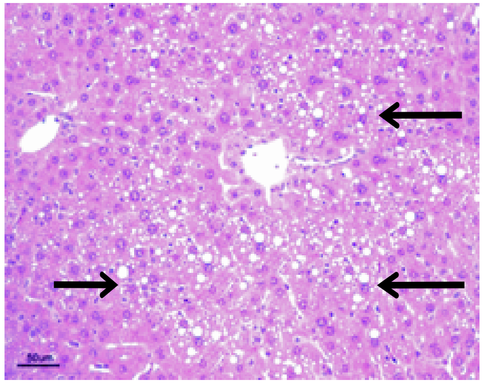

The hepatocytes of mtDNA mutator mice exhibited moderate cytoplasmic vacuolation (arrows).

C. Kidney morphology

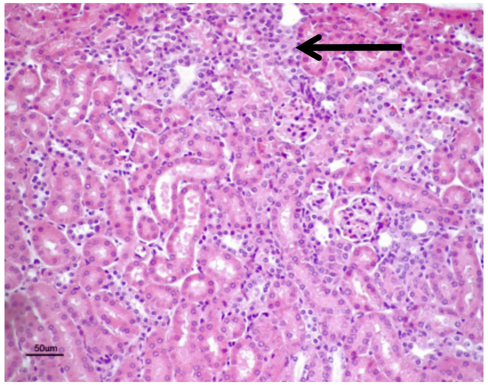

Tubular epithelial cells display increased cytoplasmic acidophilia in subcapsular areas of the cortex (arrows).

D. Scoring of paleness

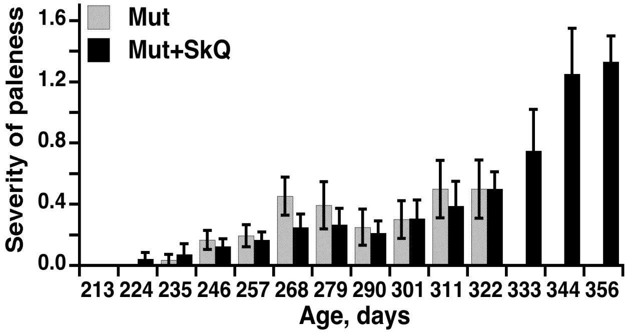

Supplementary Figure S2 (related to Table 3). Necropsy and histopathological findings in SkQ1-treated or non-treated mtDNA mutator mice.

Pathologies not affected by SkQ1 treatment

Gross changes at necropsy of both treated and non-treated mtDNA mutator mice consisted of emaciation, thin coat, body pallor, fat atrophy, and pale liver and enlarged spleen, suggestive of anemia. The gastrointestinal tract with its content was normal except for the cecum that was of reduced size with pasty, dark green dehydrated contents that were harder than normal. Extensive mucosal hyperplasia with sites of inflammation, microabscesses (not particularly associated with any specific microorganism) observed in cecum and proximal part of colon were main findings in all aged mtDNA mutator mice. In some mice, a swollen anus with slight eversion of the rectal mucosa consistent with rectal prolapse was observed. Extensive extramedullary hematopoiesis in spleen red pulp and scattered hematopoietic cells in hepatic sinusoids were observed in many mtDNA mutator mice.

Pathologies affected by SkQ1 treatment

Several other pathological changes in tissues (i.e. heart, liver, kidney, skeletal muscles) of mtDNA mutator mice were significantly attenuated by SkQ1 treatment. In heart and skeletal muscles, SkQ1 attenuated myocardial degeneration (**A**) and sarcopenia.
The pathological changes observed in the liver of mtDNA mutator mice were of three types:

1) the sinusoids exhibited scattered hematopoietic cells consistent with severe anemia;
2) the hepatocytes displayed rich microvesicular vacuolization, (fatty changes) consistent with hepatic steatosis (representative finding shown on **B**);
3) degenerative changes with abnormal nuclear features (pseudo-inclusions) typical for aging.

Long-term treatment of mtDNA mutator mice with SkQ1 significantly reduced the frequency of the appearance of hepatic steatosis and these degenerative changes.
Common findings in kidney were mild-moderate tubular and interstitial changes in the renal cortex consistent with nephropathy (**C**), which were significantly attenuated by SkQ1 treatment.
